# Tumefactive Multiple Sclerosis Variants: Report of Two Cases of Schilder and Balo Diseases

**Published:** 2017

**Authors:** Mahmoud Reza ASHRAFI, Ali Reza TAVASOLI, Houman ALIZADEH, Javad ZARE NOGHABI, Nima PARVANEH

**Affiliations:** 1 Department of Pediatrics, Division of Pediatric Neurology, Children’s Medical Center, Tehran University of Medical Sciences, Tehran, Iran; 2 Department of Pediatrics, Division of Pediatric Radiology, Children’s Medical Center, Tehran University of Medical Sciences, Tehran, Iran; 3 Department of Pediatrics, Ardabil University of Medical Sciences, Ardabil, Iran; 4 Department of Pediatrics, Division of Allergy and Clinical Immunology, Children’s Medical Center, Tehran University of Medical Sciences, Tehran, Iran

**Keywords:** Central Nervous System, Demyelination, Tumefactive Lesion, Multiple Sclerosis Variants, Schilder Disease, Balo Disease

## Abstract

A tumefactive lesion of central nervous system (CNS) is defined as a mass-like lesion with a size greater than 2 cm in brain detected by magnetic resonance imaging (MRI). Neuroimaging may help to distinguish the nature of a tumefactive lesion and therefore, can prevent an unnecessary brain biopsy. Here we emphasized on determining the nature of a CNS tumefactive lesions with the help of MRI and more explanations about demyelinating lesions with focus on Schilder and Balo diseases as two multiple sclerosis variants. We have reported here two boys of 10 and 8 years of age respectively of multiple sclerosis (MS) variants who presented with acute neurologic complications to our hospital as one of the two referral children hospital in Tehran, Iran. Tumefactive demyelinating lesions can be considered a separate entity that itself can contain Schilder disease, Balo disease, some cases of acute disseminated encephalomyelitis (ADEM) or classic MS. MRI can help to establish a diagnosis of a tumefactive lesion and to differentiate among different underlying etiologies.

## Introduction

Magnetic resonance imaging (MRI) of the central nervous system (CNS) is essential for initial investigation of a child presented with acute or sub-acute focal neurologic problems, which may show a mass-like lesion. The four differential diagnoses of a tumefactive (mass-like) CNS lesion found on MRI are tumors and infiltrative lesions, vascular lesions, abscess/CNS parasitic infections and finally acquired demyelinating disorders (1, 2).

Demyelinating diseases of the nervous system are divided into two major groups based on anatomical localization: demyelinating diseases of CNS and demyelinating diseases of peripheral nervous system (PNS). The major entities in the spectrum of CNS demyelinating diseases are classic multiple sclerosis (MS), MS variants (Schilder, Balo, Devic and according to some theories Marburg disease), acute disseminated encephalomyelitis (ADEM), acute hemorrhagic leukoencephalitis (Hurst Disease), optic neuritis, acute transverse myelitis (ATM), and acute cerebellar ataxia (ACA) (3). The most important disorders categorized in PNS demyelinating group are Guillain-Barré syndrome (GBS) and chronic inflammatory demyelinating polyradiculoneuropathy (CIDP). Features such as clinical presentation at disease onset, age at onset, course of the illness, number and location of the lesions in the CNS imaging, previous medical or vaccination history and cerebrospinal fluid (CSF) characteristics, can help us to distinguish different types of these disorders (3). 

Some cases of classic MS, MS variants such as Schilder or Balo disease and some cases of ADEM are the most important acquired demyelinating disorders that can produce tumefactive appearance in brain MRI. 

Simply, the presence or absence of fever and signs of encephalopathy, previous history of URI (upper respiratory infection) or vaccination as well as brain imaging finding scan help us to differentiate between ADEM and MS variants (3-8). Schilder and Balo diseases are rare MS variants that cause CNS demyelinating disorders. Since the histopathological features of these diseases are similar to MS, they are classified as MS variants, although their clinical features are different from MS to some degree (5, 6). Schilder and Balo diseases may look resembling in terms of clinical symptoms with each other, but neuroradiologic and pathologic features help to differentiate between them. 

Correct diagnosis of the nature of a tumefactive lesion is important, preventing unnecessary brain biopsy, surgery or therapeutic procedures such as radiotherapy or chemotherapy (9). Nowadays, fortunately, different neuroradiologic methods help us to determine the nature of these lesions and in some cases; they eliminate the need for invasive procedures such as brain biopsy (4,10). 

Here, we emphasized the MRI finding of Schilder and Balo diseases presenting two real cases.

## Case Report

Two patients (two boys with 10 and 8 years old) presented to our hospital as one of the two big referral children hospital in Iran with acute and subacute neurologic complications. Further medical evaluation revealed onset of acute tumefactive demyelinating disease in these two cases. We report clinical and laboratory findings of these two patients in details with a brief review of the role of neuroimaging modalities in how to manage tumefactive lesions in brain MRI.

Consent form was written and filled by biologic parents of both two patients to include in our study.

**Patient 1**

The first case was a 10-yr-old boy with normal development referred to our hospital with sub-acute onset of right-circumduction gait, some degree of vision loss, expressive aphasia, vomiting and headache started from 1 month ago. There was no history of recent URI or vaccination. In physical examination, he had no fever but right foot drop and right arm weakness in association with bilateral downward plantar reflexes. Fundoscopic examination of the patient showed bilateral papilloedema (grade 2-3). Routine blood chemistries were normal. 

Brain computed tomography CT scan had been performed elsewhere helped to implement CSF puncture unwarily.

CSF analysis showed normal degree of protein, glucose, IgG index, lactate, oligoclonal band (CSF OCB) and negative AQP4 antibody. CSF opening pressure was about 28 cm H2O. Bacterial and viral assessments of CSF and CSF polymerase chain reaction PCR for tuberculosis were all negative. Serum very long-chain fatty acid (VLCFA) levels and adrenal function tests were normal. 

**Fig 1.**
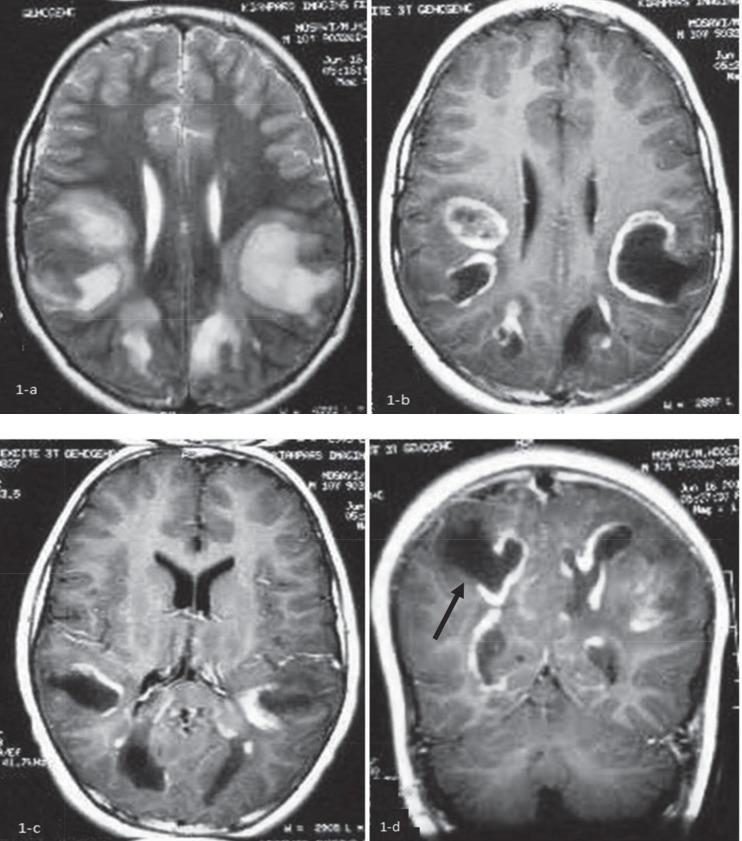
Initial brain MRI without contrast shows bilateral abnormal high signal lesions in the subcortical and deep posterior white matter in T2-WI sequence, almost isointense with CSF in the center, surrounded by a peripheral halo (a): After contrast injection In T1-WI images, low signal center with peripheral complete and incomplete ring enhancement is seen, suggesting necrotic or tumefactive center (b, c, d). Some cortical extension of the lesion in the right posterior parietal lobe is seen (Black arrow in d

**Fig 2:**
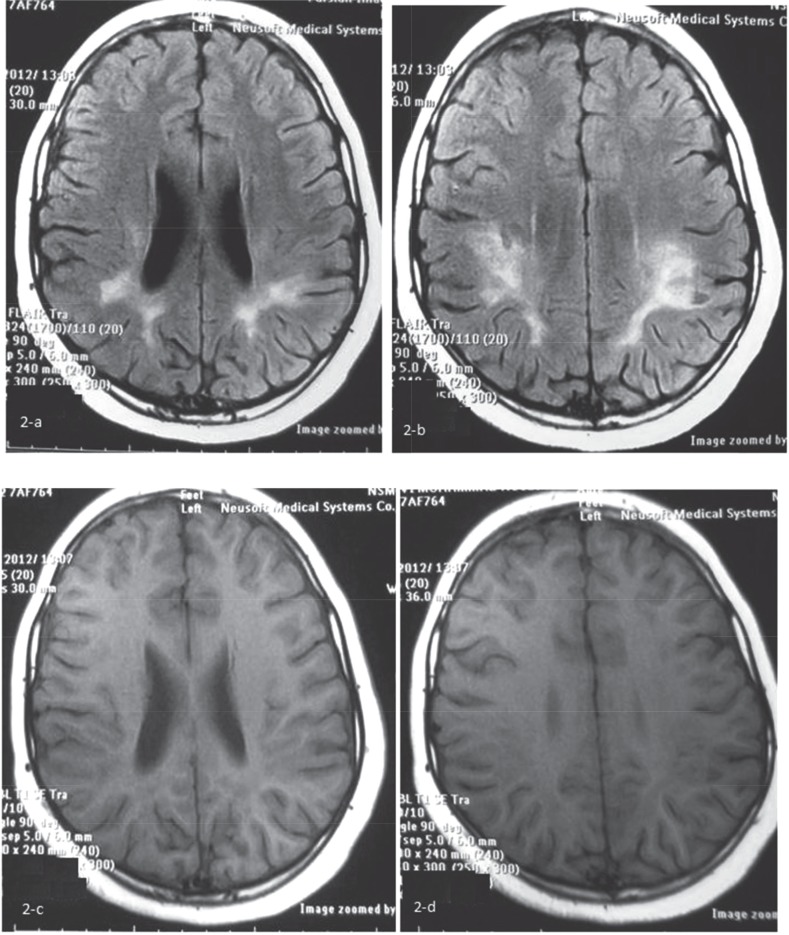
Brain MRI with contrast showed remaining hyper signal in T2- FLARE from sequel of treated demyelinating lesions (a,b) and just disappearance of tumefactive lesions in T1-WI (c,d)

Initial brain MRI without contrast showed bilateral abnormal high signal lesions in the subcortical and deep posterior white matter in T2-WI sequence that was almost isointense with CSF in the center, surrounded by a peripheral halo (Figure 1a). In post contrast, T1-WI image show signal lesions with peripheral complete and incomplete ring enhancements were noted, suggesting necrotic or tumefactive center. Some cortical extension of the lesion located in the right posterior parietal lobe was remarkable (Figure 1b-d). The clinical course, atypical presentation, relative high opening pressure of CSF and radiologic findings in brain MRI with contrast suggested the probable diagnosis of the Schilder disease. The patient was treated with a 5-day course of methylprednisolone pulse (20 mg/kg/d). His clinical condition was bettered after 10 d and due to some neurologic residue such as hemiparesis and vision problem, oral prednisolone was continued for about 4 months (1-2mg/kg) and then tapered. The patient was followed for about 2 yr and all of his symptoms was recovered, completely. He showed no disease flare-up during the follow-up period. We do not perform brain biopsy and MRS (magnetic resonance spectroscopy) for him and brain MRI with contrast after 2 yr of the onset of the disease showed just remaining residual hypersignal lesions in T2- FLAIR as a sequel of treated demyelinating lesions (Figure 2a,b) and disappearance of tumefactive lesions in T1-WI (Figure 2c, d). 

**Patient 2**

**Fig 3:**
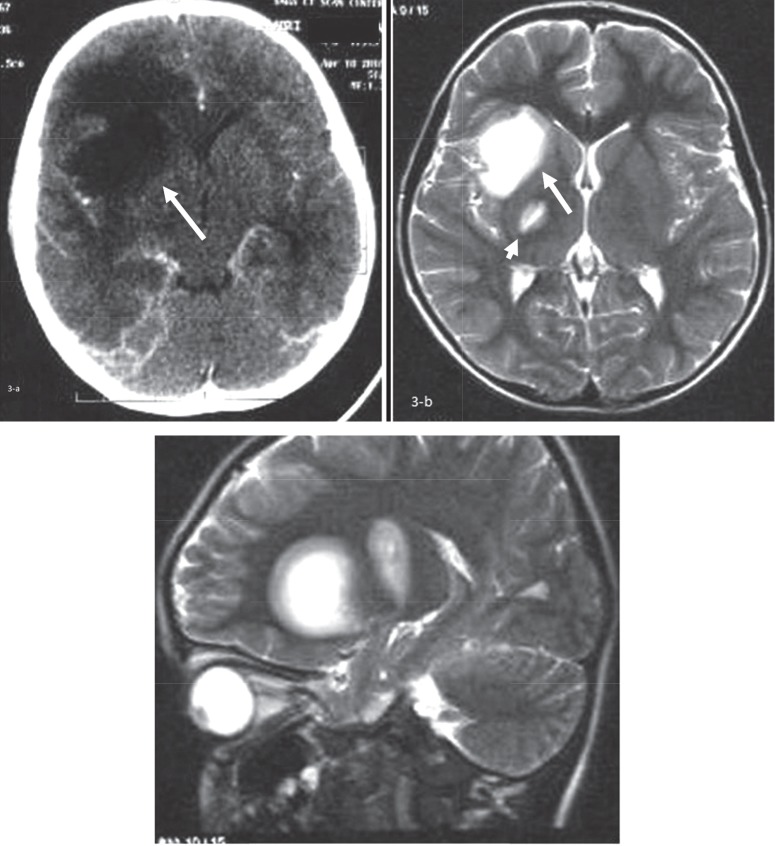
Brain CT scan with contrast demonstrated low attenuated subcortical areas in the right frontal and right lentiform nucleus without significant mass effect or post contrast enhancement (a-White arrow). The first brain MRI of the patient showed a couple of hypersignal lesions in T2-WI. The larger one is located in the right lentiform nucleus and insular subcortical white matter with surrounding halo (Large white arrow-b). The smaller one was located in the posterior limb of right internal capsule (Small white arrow-c

The second case was an 8-yr-old boy with normal development who presented with acute onset of left-sided hemiparesis of the face and subsequently weakness of left-side limbs following a mild head injury. He had no history of recent viral illnesses or vaccination. Physical examination revealed no loss of consciousness or fever, but he had left-sided limb paresis with hyperreflexia and central left facial nerve palsy. Other neurologic examinations were unremarkable. Brain CT scan with contrast demonstrated low attenuated subcortical areas in the right frontal lobe and right lentiform nucleus with some mass effect (Figure 3a).

The first brain MRI of the patient showed a couple of hypersignal lesions in T2-WIsequence. The larger one was located in the right lentiform nucleus and insular subcortical white matter with surrounding halo. The smaller one was located in the posterior limb of right internal capsule (Figure 3b, 3c). Lumbar puncture revealed CSF protein of 10 mg/dl, negative oligoclonal band and AQP4 antibody and no CSF pleocytosis.

Virology assessment of CSF for HSV, VZV and HIV were all-negative. The patient was treated with a 4-day course of methylprednisolone pulse with presumptive diagnosis of Balo disease. The clinical symptoms ameliorated, but due to remaining some degree of leftsided limbs weakness, physical therapy was started. 

**Fig 4:**
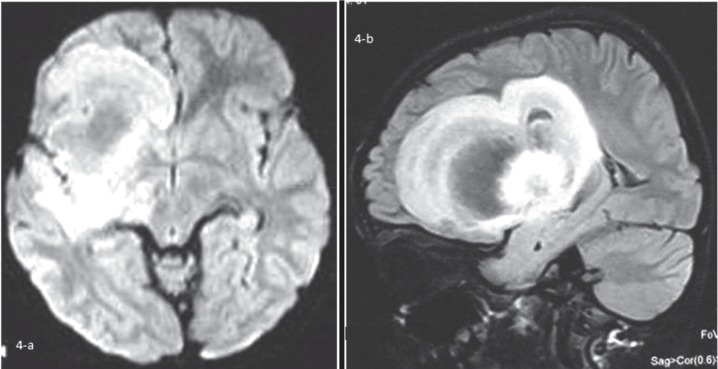
The second brain MRI revealed very large mass like concentric or layered high signal lesion in FLARE sequence in the right frontal lobe white matter and adjacent basal ganglia

About 2 months later, he presented to the Emergency Department with an attack of a febrile seizure. After doing brain CT-scan, the second brain MRI was done that revealed very large mass like concentric or layered high signal lesion in FLAIR sequence in the right frontal lobe white matter and adjacent basal ganglia (Figure 4a, b).

A brain biopsy of the right frontal lesion was done and showed a lesion with macrophage infiltration, prominent gemistocytic gliosis and no evidence of malignancy. 

These findings were compatible with a demyelinating disease and according to neuroradiologic findings, the diagnosis of Balo concentric sclerosis was considered. 

**Fig 5:**
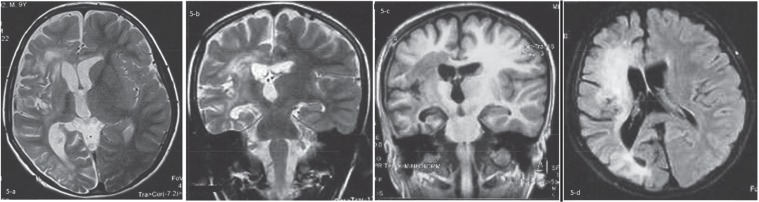
The third brain MRI has done 6 months after biopsy, demonstrated volume loss in the right hemisphere with secondary ventricular prominence. Mass like lesion in the second brain MRI in the right frontal lobe could not be seen any longer (a,b) and abnormal periventricular white matter signal would suggest diffuse gliotic changes or less likely sequel of previous demyelinating lesion (c,d

Carbamazepine was started for the patient and his clinical course followed for 2 yr. The third brain MRI had done 6 months after biopsy, demonstrated volume loss in the right hemisphere with secondary ventricular prominence. Mass-like lesion seen in the second brain MRI in the right frontal lobe could not be seen any longer and abnormal periventricular white matter signal would suggest diffuse gliotic changes or less likely sequel of previous demyelinating lesion (Figure 5a-d). The last brain MRI (2 yr after biopsy) had not any more changes as compared with third brain MRI (not shown here). The patient had no flare-up of similar symptoms or seizure recurrence during these 2 years of follow-up. 

## Discussion

Normally, tumefactive demyelinating lesion is defined as a mass-like lesion with a size greater than 2 cm in brain MRI. Its radiologic features may include mass effect, local edema, ring enhancement and decreased relative cerebral blood volume (4, 9, 11, 12). However, tumefactive demyelinating lesion was occasionally defined as a lesion with a diameter of greater than 3 cm (13). Sometimes, these lesions make a diagnostic uncertainty because they may be confused with an intracranial tumor. Other two important differential diagnoses of a tumefactive lesion in brain MRI in children are abscesses and vascular malformations (2). 

Tumefactive demyelinating lesions occur mainly in cerebral hemispheres but may be seen in other parts of CNS, such as cervical spinal segments (9,14). Between various types of demyelinating diseases, ADEM, some cases of classic MS and MS variants such as Schilder and Balo disease can present themselves with a pattern of tumefactive lesion in the brain MRI (9). 

The onset of neurologic symptoms of tumefactive demyelinating lesions may be acute or subacute (9, 10). These symptoms can include headache, vomiting, cognitive and behavioral changes, increased intracranial pressure, visual disturbance, optic neuritis, deafness, hemiparesis and or seizure (3,7,9). The first case (Schilder disease) presented with signs of increased Intra Cranial Pressure (ICP), hemiparesis and some degree of visual loss and speech problem (expressive aphasia). 

One of interesting signs in this patient less reported is speech problem. In addition, the second case (Balo disease) showed hemiparesis and seizure in his clinical course. Increased ICP have been reported in Schilder disease (3). 

Schilder and Balo diseases are two rare demyelinating disorders of CNS that may have some similarity in clinical symptoms with each other, but neuroradiologic and pathologic findings help us to distinguish them from each other. The original case of Schilder disease was described by Schilder in 1912 but in 1986 Poser established revised diagnostic criteria for non-invasive diagnosis of true Schilder disease (2,4). Neuropathologic features of this disease that affect predominantly children or young patients are sharply demarcated giant coalescent plaques of demyelination that involve bilateral cerebral hemispheres. Imaging shows bihemispheric lesions of white matter with incomplete ring enhancement and some degree of edema around the lesions that do not involve subcortical U fibers (unlike ADEM) even in severe cases (2-4, 7, 14). Furthermore, in Schilder disease, unlike ADEM usually there is no history of recent viral illness or vaccination (15). The mainstay of its treatment is high dose of steroid that makes usually good clinical response. However, in one patient successful treatment with IVIG also has been reported (3,7). On the other hand, Balo disease mainly occurs in young adults and is more common in males (16). Its clinical course may be more severe than Schilder disease; although some cases of Balo disease with benign course have been reported (16-18). Neuropathologic features consisting of concentric rings of demyelination, alternating with rings of normal white matter and imaging reveals lamellar and concentric pattern on proton and T2-weighted brain MRI images with alternating bands of enhancement and non-enhancement (19, 20).

**Table 1:** Characteristic Features of Brain Tumefactive Lesion (Its Differential Diagnosis) in Multiple Neuroradiologic Measures (22-27)

**Type of lesion**	**T2-WI**	**T2-FLAIR**	**T1-WI**	**T1-WI with Gad**	**Restriction in DWI**	**ADC**	**PWI**	**MRS**
**Acute or subacute tumefactive demyelinating lesion**	Generally High signal in the center and periphery	Could be low signal in the necrotic center	Low Signal	Complete or incomplete ring enhancement	+/-	↑	Generally, increased rCBV in the periphery of lesions with severe inflammatory reaction	↓NAA,↑↑choline, ↑lactate,↑lipid ↑glutamate/ glutamin, ↑myo-inositol
**Primary neoplastic lesions of CNS**	High signal	Low signal in the necrotic center	Low signal	+/- If enhanced, Solid components or ring pattern	+/-	↑	Increased rCBV in the periphery of lesion(higher than abcess)	↓NAA,↑↑choline, ↑lactate,↑lipid, Unchanged Aminoacid, ↑acetate, ↑alanine, ↓succinate
**Abscess**	High signal with severe peripheral vasogenic edema	Low signal in the necrotic center	Low signal	Complete ring enhancement	++	↓	Decreased rCBV in the periphery of lesion	↓NAA,↑Lactate, ↑choline, ↑lipid ↑acetate, ↑succinate ↑Aminoacid, ↑alanine↓creatine
**CNS vasculitis**	High signal	Low signal in the necrotic center	Low signal	+/-	++	↓	Usually unchanged rCBV	↓NAA,↑choline slightly↑lactate, ↑↑glutamate/ glutamine,slightly ↑lipid

Radiologic features of the first case in brain MRI were compatible with Schilder disease but we did not perform brain biopsy for neuropathologic evaluation. Based on criteria, histopathological evidence is necessary for diagnosis of Schilder disease (10). In recent years, the possibility of the diagnosis of this disease has been proposed by means of neuroradiologic measures in some reports because brain biopsy is not diagnostic in about 20% of patients and it is associated with some dangers such as hemorrhage (4,10). This case had good clinical response to high-dose of methylprednisolone and oral prednisolone was continued and tapered off during 4 months until full improvement of clinical condition of the patient. In the second case, neuropathologic and radiologic features were compatible with Balo disease. 

In this case, brain biopsy was done initially with probable diagnosis of brain tumor. The pathologic study of brain biopsy of this patient confirmed the diagnosis of Balo disease.

It is worth determining the nature of tumefactive demyelinating lesions (TDL) by means of noninvasive diagnostic tools, mainly neuroradiologic tools. 

Neuroimaging may help to prevent brain biopsy that is an invasive measure and is associated with some morbidity (0.7%-9%) and mortality (0%-1.2%) (10,14). 

In the second case, it was possible to reach the diagnosis of demyelinating lesion without brain biopsy, especially that the clinical symptoms of the patient ameliorated with trial course of steroid and the size of the lesions were decreased in the following brain MRI (compare Fig 4 and 5).

Different neuroradiologic measures can help us to characterize the nature of a TDL in brain MRI in a patient with focal neurologic deficit (Table 1) (21-26). 

These techniques in addition to brain MRI include MRS (Magnetic Resonance Spectroscopy), diffusion- weighted imaging (DWI) and perfusion-weighted imaging (PWI), apparent diffusion coefficient (ADC). 

Another way that can help to distinguish demyelinating nature of a tumefactive lesion from another differential diagnosis such as an abscess or neoplasm is good clinical response to steroid and reduction of the size of the lesions in serial neuroimaging (14).

**In conclusion, **demyelinating diseases of childhood have a wide clinical spectrum that can be divided into two groups that involve peripheral or central nervous system (3,5). We suggest among those who involve CNS, TDL can be considered a separate entity that itself can contain Schilder disease, Balo disease, some cases of ADEM or classic MS (27). In the absence of a biological marker, distinguishing of these disorders from each other can sometimes be difficult, but clinical course and use of neuroradiologic measures can help to establish a diagnosis and to differentiate them from other causes. 

## Author’s Contribution

Ashrafi MR: Designing, Final approval of the work 

Tavasoli AR: Drafting, Interpretation of the data, final approval of the work

Alizadeh H: Neuroimaging interpretation

Zare Noghabi J: Drafting, Refer one of the two patients

Parvaneh N: Draft, Critical review

All authors agreed to be accountable for all aspects of the Work in ensuring that questions related to the accuracy or integrity of any part of the work is appropriately Investigated and resolved.
